# Bayesian network prior: network analysis of biological data using external knowledge

**DOI:** 10.1093/bioinformatics/btt643

**Published:** 2013-11-09

**Authors:** Senol Isci, Haluk Dogan, Cengizhan Ozturk, Hasan H. Otu

**Affiliations:** ^1^Bogazici University, Institute of Biomedical Engineering, Kandilli Campus, 34684, Cengelkoy - Istanbul, ^2^TUBITAK-BILGEM, Informatics and Information Security Research Center, 41470, Gebze-Kocaeli and ^3^Istanbul Bilgi University, Department of Genetics and Bioengineering, 34060, Eyup - Istanbul, Turkey

## Abstract

**Motivation:** Reverse engineering GI networks from experimental data is a challenging task due to the complex nature of the networks and the noise inherent in the data. One way to overcome these hurdles would be incorporating the vast amounts of external biological knowledge when building interaction networks. We propose a framework where GI networks are learned from experimental data using Bayesian networks (BNs) and the incorporation of external knowledge is also done via a BN that we call Bayesian Network Prior (BNP). BNP depicts the relation between various evidence types that contribute to the event ‘gene interaction’ and is used to calculate the probability of a candidate graph (G) in the structure learning process.

**Results:** Our simulation results on synthetic, simulated and real biological data show that the proposed approach can identify the underlying interaction network with high accuracy even when the prior information is distorted and outperforms existing methods.

**Availability:** Accompanying BNP software package is freely available for academic use at http://bioe.bilgi.edu.tr/BNP.

**Contact:** hasan.otu@bilgi.edu.tr

**Supplementary Information:** Supplementary data are available at *Bioinformatics* online.

## 1 INTRODUCTION

Gene interaction (GI) networks provide insight for understanding the biological mechanisms that explain various phenotypes in health and disease. The inference of GI networks from high-throughput biological data is an important and challenging task in systems biology. Throughout the literature, the term GI has been used in a broad sense implying direct and indirect interactions between genes and/or gene products. Several machine learning and statistical methods have been proposed for the problem (Akutsu *et al.*, 2000; D’Haeseleer *et al.*, 2000; Hecker *et al.*, 2009; Lezon *et al.*, 2006; Liang *et al.*, 1998; Yeung *et al.*, 2002) and Bayesian network (BN) models have gained popularity for the task of inferring gene networks (Friedman and Koller, 2003; Friedman *et al.*, 2000; Hartemink, 2005; Kim *et al.*, 2003). Because of the complexity of GI networks and the sparse, noisy nature of experimental data, machine learning and statistical methods may lead to poor reconstruction accuracy for the underlying network. One way to overcome this problem would be to incorporate prior biological knowledge when making network inferences using experimental data. Due to technological advances in sequencing, microarray, proteomics and related fields, biological and clinical data are being produced at an ever increasing rate. The 2013 database issue of the *Nucleic Acids Research* journal lists 1512 molecular biology databases, which provide a vast amount of annotated data and meta data that could be used in a systematic way (Fernandez-Suarez and Galperin, 2013).

BNs have a number of features that make them viable candidates for combining prior knowledge and data as BNs can deal with uncertainty, avoid over fitting a model to training data, and learn from incomplete datasets. BNs handle stochastic events in a probabilistic framework accounting for noise, which results in emphasizing only strong relations in the observed data. Furthermore, BNs are able to focus on local interactions where each node is directly affected by a relatively small number of nodes (Friedman *et al.*, 2000) and interactions defined by a BN can be related to causal inference (Verma and Pearl, 1991). These properties are similarly observed in biological networks justifying the use of BNs in exploring pathways in the setting of identifying GI networks using experimental data. Learning algorithms for both the structure and parameters of BNs have been developed (Neapolitan, 2004). Most of the research on BNs has focused on directed acyclic graphs (DAGs) and static systems with discrete variables and/or linear Gaussian models. Friedman *et al.* (2000) used BNs to generate a causal model of the yeast cell-cycle data using either a model with discretized expression levels (e.g. Boolean, or underexpressed/normal/overexpressed), or a linear Gaussian model. The latter treats the expression level of a gene as being normally distributed around a mean which is a linear sum of inputs. Therefore, rather than true causal relationships, the results may represent co-regulation of genes. Accordingly, a method to sample network structures from the posterior distribution with Markov Chain Monte Carlo (MCMC) has been introduced (Friedman and Koller, 2003).

Many BN structure learning algorithms are based on heuristic search techniques with the likelihood approximation because of the infeasible computational complexity. These approaches may lead to a false model, as neither the search technique nor the objective functions guarantee the optimal solution. Informative priors generated from existing biological information can improve structure learning to get better models to describe the underlying GIs. In several studies the use of prior biological knowledge in conjunction with gene-expression data has been shown to improve the fidelity of network reconstruction. Hartemink *et al.* (2002) incorporated genomic location data to guide the BN model inference. Tamada *et al.* (2003) proposed a method, which iteratively detects consensus motifs based on the structure of the estimated network model, then evaluates the network using the result of the motif detection, until the inferred network becomes stable. Imoto *et al.* (2003) proposed a framework utilizing Gibbs distribution where an energy function was used to evaluate the probability of an edge in the inferred networks. Werhli and Husmeier (2007) extended this approach to integrate multiple sources of prior knowledge into dynamic Bayesian network (DBN) learning via MCMC sampling. Mukherjee and Speed (2008) proposed a scheme to incorporate known network features including edges, classes of edges, degree distributions, and sparsity into gene network reconstruction within a Bayesian learning framework utilizing MCMC sampling.

These studies were limited in the use of external biological knowledge by incorporating only certain features, such as network topology or binding sites in promoter regions. Furthermore, in the aforementioned approaches manual curation and/or incorporation of the external knowledge are employed. In this article, we present a framework to incorporate multiple sources of prior knowledge, regardless of its type, into BN learning. The meaning of prior knowledge in our context is the enumeration of pair-wise interactions of genes from biological information sources and the use of this information in BN modeling. The proposed method is fully automatic and does not use likelihood approximations to find the optimal network that explains observed experimental data. We propose a novel framework that uses BN infrastructure itself to incorporate external biological knowledge when learning networks. This infrastructure yields GI information for pairs of genes, which can be used as informative priors to calculate the probability of a candidate graph, G. This information is then incorporated in the network-learning process that tries to identify the most probable graph given data. We provide an open-access web-based implementation of the proposed method at http://bioe.bilgi.edu.tr/BNP. Our results indicate that we can successfully reconstruct networks using synthetic data in addition to simulated and real gene-expression data.

## 2 METHODS

**Fig. 1.**
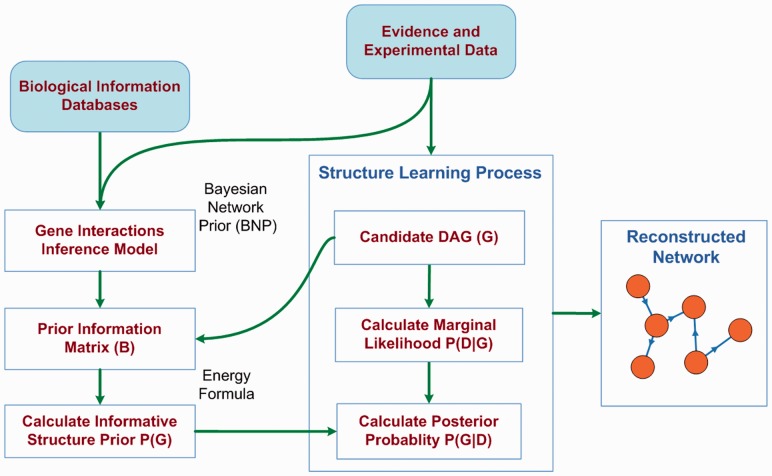
Overall workflow of the proposed method. BNP is constructed using GI information from external biological databases and when instantiated with an evidence vector for a pair of genes, the GI probability is inferred. For a list of genes, the pair-wise interaction information is stored in the prior matrix *B*, which is used to calculate the probability of a candidate graph *G* in the structure learning process

The schematic depiction of the overall proposed method is presented in Figure 1. Pair-wise interaction information is gathered from biological databases and a BN model for prior knowledge, Bayesian Network Prior (BNP) is developed. In BNP, one node is depicted as GI and the topology represents the dependence structure for different evidence types within each other and with the GI node. For a set of genes, the model is instantiated with the given evidence and/or experimental data for each pair of genes. The GI node is used to infer whether the gene pair is related or not, represented by a prediction value between 0 and 1. A prior knowledge matrix, *B*, is populated with these prediction values for all gene pairs. Using a proposed novel energy formula and informative prior formula, this prior knowledge is utilized to calculate the probability of a candidate DAG, G, in the structure learning process. This parameter is used to optimize *P*(*G*|*D*) instead of the likelihood, *P*(*D*|*G*), used by existing structure learning algorithms.

### 2.1 Informative structure priors

A BN is a compact graphical representation of the joint probability distribution over a set of random variables and consists of a DAG = (*V*, *E*), with a node set *V* corresponding to the random variables *X*_1_, … , *X_n_* and an edge set E on these nodes and a set of conditional probability distributions Θ for each node in the DAG. The DAG encodes the assertions of conditional independence. If the random variables are discrete, conditional probability distributions Θ can be represented as a set of conditional probability tables (CPT). CPTs list the probabilities for each value that a child node can assume given a combination of values of its parents.

In GI network-modeling studies using BNs, *X_i_* represents a gene and edges represent relationship between genes. The task of network inference (i.e. structure learning) is to make inferences regarding the graph G that best explains the data. This can be achieved by finding the DAG G that maximizes

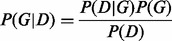

where *P*(*D*|*G*) is the likelihood, *P*(*D*) is the probability of the data, *P*(*G*) is the structure prior (or network prior) probability of the graph *G* and *P*(*G*|*D*) is the posterior probability of *G*. In commonly used heuristic structure learning algorithms, *P*(*D*|*G*) is optimized instead of the true model *P*(*G*|*D*). The likelihood criterion does not guarantee to find the optimum solution even if a heuristic approach is not employed. Nevertheless, optimizing the likelihood can be justified by assuming *P*(*D*) and *P*(*G*) to be equal for all *G*. The former assumption can be regarded as reasonable as *D* is observed. However, the latter assumption is generally not correct and is made mainly due to difficulties in calculating *P*(*G*) and/or lack of prior knowledge on *G*. Use of uniform (flat) priors for *G*s ignores the contribution of *P*(*G*) and this may cause failure in differentiating between DAGs that are in the same Markov equivalence set. Therefore, the true DAG among the ones that support the same conditional probability distribution cannot be identified. The proposed approach aims to calculate P(G) using external knowledge and provide improvements in the structure-learning phase for GI networks.

For discrete BNs, most of the learning tasks are performed by calculating *P*(*D*|*G*) with the Bayesian Dirichlet equivalent (BDe) scoring function and by assuming uniform (flat) prior structure for all possible candidate DAGs (Heckerman *et al.*, 1995). In the proposed approach, we employ a greedy search algorithm that aims to maximize *P*(*G*|*D*). For a given candidate DAG, *G*, we calculate *P*(*G*) by first obtaining the prior information matrix, *B*. Unlike existing methods, the proposed approach does not use categorized prior knowledge but assigns probabilities to each candidate edge. The matrix B is obtained by instantiating BNP with the evidence vector for each pair of genes in the input gene set. These evidence vectors can originate from any performed experimental data at hand, or external knowledge, or both.

Let *B* be the prior information matrix, where *B*(*i*, *j*) = *P*(*X_ij_*), the degree of prior belief that gene *i* and *j* interact based on external knowledge. Let *A_G_* denotes the adjacency matrix of the candidate graph *G*. We define the matrix *U* such that *U*(*i*, *j*) = 1 – [*B*(*i*, *j*)*A_G_*(*i*, *j*)], the element by element multiplication of *B* and *A_G_*. Note that if there exists no edge from *i* to *j* in *G*, *U*(*i*, *j*) = 1; and if there is an edge from *i* to *j* in *G*, *U*(*i*, *j*) is inversely proportional to our prior belief on the existence of the edge. The total energy of *G* is defined as:

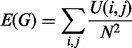

where *N* is the number of nodes in *G*. This way, we do not assign categorical values for *U*(*i*, *j*) and exploit fully the information about prior existence of an edge. Informative structure prior is formulated as:

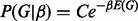

where *C* is a scaling constant. The choice of *C* does not affect the relative comparison during scoring of graphs in structure learning. The hyperparameter *β* can be marginalized using

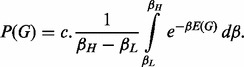



For ease of simulation, the integral is calculated for a range of *E*(*G*) and stored in a lookup table. In the numerical calculation of this integral, Δ*β* = *β_H_* – *β_L_* is the parameter of interest, which is optimized as explained in subsection 3.2. The integration approach automatically incorporates the uncertainty on the parameter by averaging the likelihood values of the parameter. Point estimates acquired by maximizing the parameters may change arbitrarily with arbitrary re-parameterizations. Point estimates maximize the probability density without taking into account the complementary volume information, which may yield in suboptimal results. When one has a choice of which variables to integrate over and which to maximize over, it is suggested that one would integrate over as many variables as possible in order to capture the relevant volume information of high-dimensional probability distributions (MacKay, 1996, 1999).

### 2.2 Bayesian network prior

The goal in building BNP is to construct a framework such that the distilled external biological knowledge is used in an intelligent way to make an assessment about the interaction of a pair of genes. Previously, Troyanskaya *et al.* (2003) proposed a Bayesian Framework for combining various data sources for gene function prediction. In this method a Naive Bayesian model was constructed. The parameters (CPTs) of the model were determined by experts. Then, a separate network was instantiated for each gene pair by initializing the bottom-level nodes with evidence and the probability of the functional relationship between the two genes was updated. The model was designed for functional prediction, not for GI network learning. Here, we describe a novel-prior knowledge inference model that automatically learns parameters of the nodes used in BNP that predicts if two genes interact using external biological knowledge. The model organism chosen for BNP was human and the external data came from pathway, microarray, gene and protein interaction databases. The assembled information source is made up of ‘evidence types’, each making a ‘Yes’ or ‘No’ call about the interaction of two genes and BNP is the BN that represents the relation between these evidence types and GI. In what follows, we explain the data sources in detail.

Microarray co-expression was calculated using two datasets. The first dataset aims to provide a gene atlas for the human genes and examines 79 normal human tissues with 158 samples (Su *et al.*, 2004). The second database came from the ‘Reference Database for Gene Expression Analysis’ (RefExA) that represents 70 normal human tissue samples (http://www.lsbm.org). Affymetrix Expression Console v1.1 was used to normalize the samples using the MAS 5.0 method. Probe sets with absence calls in all of the samples were omitted from further analysis. Centered Pearson correlations were calculated and 71 617 pairs of probe sets with a correlation value greater than 0.98 were passed on to be used to construct BNP. KEGG (Kanehisa *et al.*, 2012), NCI/NATURE (Schaefer *et al.*, 2009) and Reactome (Vastrik *et al.*, 2007) databases were utilized to gather pathway based evidence data. 3258 pair-wise gene relations that existed in at least two of the three pathway databases were used for further analysis. A dataset was obtained from BioGrid (Stark *et al.*, 2011) with evidence of interactions that are observed in experiments with 17 different assay types such as affinity capture and two-hybrid. BioGrid analysis revealed a total of 35 600 non-redundant pair-wise interactions. After the microarray probe set level data was regressed to the gene level and all three sources were merged, 60 950 pair-wise GIs based on 19 evidence types were obtained (see Supplementary Table S1). A GI node is appended to this evidence matrix (where rows represent gene pairs and columns represent evidence types) with a ‘true’ value if there were at least two evidence types implying interaction. BNP was built by learning both structure and parameters using Greedy Hill Climbing (Neapolitan, 2004).

## 3 RESULTS

### 3.1 Constructing the BNP

BNP was built using the GI evidence matrix that contained >60 000 pairs of genes. The model was trained and tested using a 5-fold cross validation approach, where the dataset was randomized and 80% of the data was used to train the model and 20% of the data was used to test the model. Success rate of the model with respect to the GI data label is calculated as the classification error. This procedure was repeated five times and average error values were calculated. At each time, after BNP was built with 80% of the evidence matrix using the Greedy Hill Climbing (GHC) method, the remaining 20% of the data matrix was tested by inferring the value of the GI node. This test was done through instantiation of BNP using the evidence vector of a given pair of genes. Loopy Belief Propagation inference algorithm was used for inference. If the inference value was >0.5, the GI node was taken to be ‘true’. The classification error rate for the 5-fold cross validation was 0.105 ± 0.003 implying an accuracy of ∼90% when estimating if two genes interact given external biological knowledge.

**Fig. 2.**
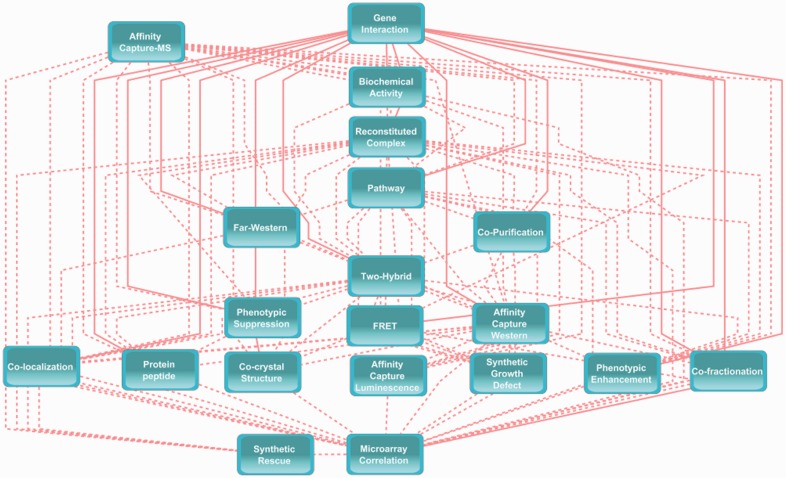
Topology of the BNP. BNP depicts the conditional dependence structure between various evidence types and the GI node based on external biological knowledge. BNP is used to predict the interaction probability for two genes using provided experimental data combined with external information. Links of the GI node are shown in solid lines for visual purposes

Final BNP was constructed with the entire evidence matrix using the GHC method. The strength of the probabilistic relationships expressed by the edges of BNP was measured using Friedman’s bootstrap method with 1000 repeats (Friedman *et al.*, 1999). Model averaging was used to build a consensus DAG of BNP, containing only the significant edges with a significance threshold of 0.413 determined by using the method of Nagarajan *et al.* (2010). The consensus DAG of BNP is shown in Figure 2. BNP consists of 20 nodes and 98 edges. The density of the network is 0.52 with an average degree of 9.8, showing high connectivity. The most connected nodes are ‘Microarray’ (denoting an interaction based on gene expression) and ‘Reconstituted Complex’ (implying an interaction is detected between purified proteins *in vitro*) with 19 edges, i.e. full connectivity. These are followed by ‘Two-Hybrid’ (18 edges), Affinity Capture MS (17 edges) and Affinity Capture Western (16 edges) assays. BNP provides a unique depiction about how different experimental assays are related to each other and to the event of GI, which opens ways to new hypotheses about assay type interrelation. The evidence matrix and the source code used to build BNP as well as the parameters of the final BNP model are available on the web portal hosting BNP.

### 3.2 Sensitivity analysis of prior parameters

We used the ubiquitous Sprinkler BN shown in Supplementary Figure S1 to test the sensitivity of the proposed method to the prior formula hyperparameter *β*. Sprinkler BN is a binary network that shows the conditional probability distributions for the events of the weather being cloudy, raining, grass being wet and the sprinkler being on. We generated simulated discrete datasets that follow the model shown in Supplementary Figure S1 using the Bayes Net Toolbox (BNT) for Matlab (http://cs.ubc.ca/∼murphyk). We used a range of Δ*β* values of [*β_L_*, *β_H_*] from 0.1 to 20 and performed receiver operating characteristic (ROC) curve analysis. The area under the curve (AUC) values for the best performing DAGs were calculated using posterior probability *P*(*G*|*D*) with informative priors (proposed method) and marginal likelihood *P*(*D*|*G*) scores with uniform flat priors. In this and all subsequent AUC calculations, the edges are considered to be undirected. For each Δ*β* value, the scoring was repeated 50 times by generating new data sizes of 10, 20, 50 and 100. In Supplementary Figure S2, we plot the mean AUC values obtained versus the parameter interval values. Our results suggest that the proposed method always outperforms the likelihood based approaches and the performance reaches a plateau for Δ*β* values of 1 and higher. Based on these observations, for the remaining experiments, we used a Δ*β* value of 10.

### 3.3 Incorporation of *P*(*G*)

Following optimization of the prior parameters, we tested the incorporation of *P*(*G*) on the Sprinkler BN as well as randomly generated 5-node BNs. In the Sprinkler BN tests, we generated data that follows the model shown in Supplementary Figure S1 for a dataset size 1000. We scored each of the 543 possible 4-node DAGs in a brute force approach without using a heuristic search algorithm. We calculated *P*(*D*|*G*) using BDe and *P*(*G*|*D*) using the proposed approach. In Supplementary Table S2, we show the top 10 scoring DAGs with the highest *P*(*G*|*D*) scores using nine different distorted prior matrix cases. Our results suggest that the proposed approach outperforms conventional structure learning methods even when the prior structure matrix *B* is vastly distorted. The true DAG comes uniquely out at the top when *P*(*G*|*D*) is considered. It is possible to differentiate between DAGs in the same Markov Equivalence Class by incorporating *P*(*G*), however, this does not hold true for the *P*(*D*|*G*) scores, which do not use *P*(*G*).

We then generated 100 random 5-node BNs along with their CPTs. Datasets of size 100 were generated with BNT and both likelihood and proposed scores were calculated for the DAGs. In applying the proposed approach, we distorted the prior matrix so that it did not always represent the true adjacency matrix. For a given DAG, we changed the real edge probabilities in the prior matrix with a fixed value between 0.7 and 1.0. This value was randomly chosen for each DAG. If no edge was present in the true DAG, this was reflected with a probability value of 0 in the prior knowledge matrix. Again a brute force method was used in that all 29 281 possible 5-node DAGs were created and scored using both methods. In Supplementary Figure S3, we show the percent rank of the true DAG for both methods with changing distortion levels. Percent rank is calculated as [(rank of the true DAG’s score/number of all DAGs) × 100%]. In all the simulations, the proposed method ranked the true DAG higher than it was ranked using marginal likelihood scoring. The average percent rank of the true DAG using the proposed method was 0.09%. In other words, the true DAG was ranked as approximately the 27th best scoring DAG, on average, using the proposed method. On the other hand, the average percent rank of the true DAG using the likelihood scoring approach was 2.28%, implying that the true DAG was ranked as approximately the 670th best scoring DAG on average.

**Fig. 3.**
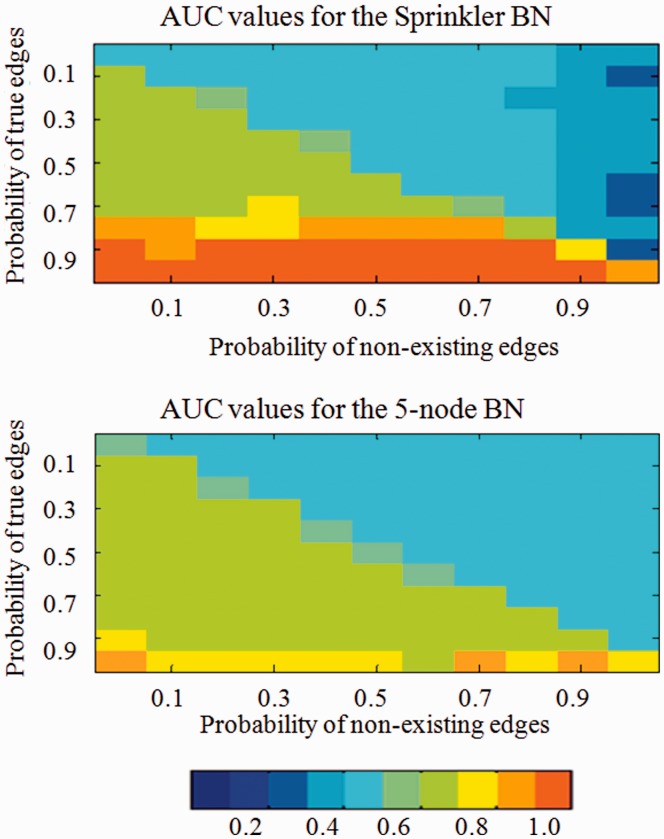
Heat map for the AUC values for the Sprinkler and a 5-node BN. The color-scale used for the heat maps are shown at the bottom. Each pixel denotes a fixed true-edge, no-edge probability pair and summarizes the mean AUC of 10 simulations. The AUC values were calculated using the proposed method based on dataset sizes of 100, each following the joint probability distribution implied by the networks. Lower left corner implies a well composed prior matrix, *B*, as true edge probabilities are close to 1 and no-edge probabilities are close to 0

We further analyzed the performance of the posterior probability scoring with informative priors against likelihood scoring with flat priors on the 4-node Sprinkler network and a randomly selected 5-node network in detail (see Supplementary Fig. S4). In the application of the proposed method, we distorted the prior knowledge matrix by assigning the same probability values, ‘*a*’, to the edges and the same probability values, ‘*b*’, to the entries with no edges using all combinations for ‘*a*’ and ‘*b*’ in the range [0,1] with 0.1 increments. The generated dataset size was 100 and the process was repeated 10 times for each pair of (*a*, *b*). In Figure 3, we show the AUC values using a heatmap. The *x*-axis represents probability values assigned to non-existing edges and the *y*-axis represents probability values assigned to the true edges in the graph. Each pixel encodes the average AUC value for the 10 simulations for a given (*a*, *b*) and the lower left quadrant represents prior knowledge that the true edge probability is in the range of [0.6–1.0] and the false edge probability is in the range of [0.0–0.4].

In the lower left quadrant, the overall mean AUC of the posterior probability scoring was >80% for the Sprinkler BN and close to 70% for the 5-node BN. If the true edges are indicated in the prior matrix with high accuracy, then the proposed method performs quite well in finding the DAG under investigation. For example, in the Sprinkler BN, when the true edges are correctly represented with a 1 in the prior matrix, AUC remains at 100% even the false edge probabilities are as high as 0.9. For a fixed true edge probability of 0.9, the average AUC is ∼92% when the false edge probability ranges from 0 to 0.9. A similar trend is observed for the 5-node BN. The heat maps shown in Figure 3 also indicate that incorrect prior knowledge is punished by our informative prior model severely and the proposed system is more robust to false positives than it is to false negatives in the prior matrix.

### 3.4 Synthetic pathway data

**Fig. 4.**
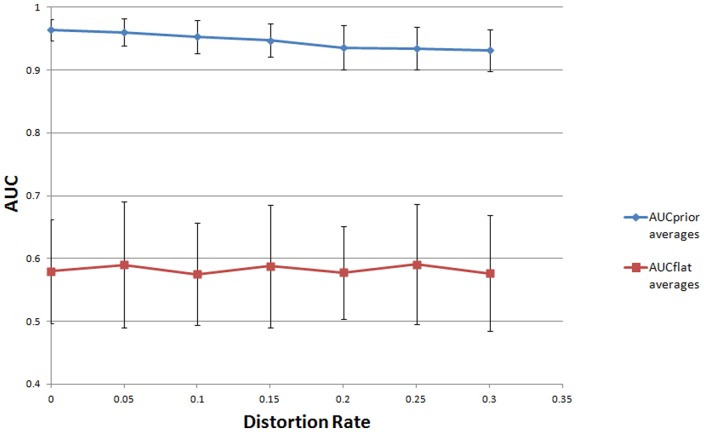
Average AUC values for the proposed (prior) and standard likelihood based (flat) methods. The *x*-axis represents the distortion rate used in the prior matrix, *B*. Twenty-three KEGG pathways were modeled as BNs and five fitting datasets of size 50 for each pathway was generated. Learned networks using the two methods were compared to the KEGG pathways for AUC calculation. In application of the proposed method the adjacency matrix of the original pathway was distorted by adding Gaussian noise to the matrix entries

We picked 23 human KEGG pathways and modeled them as BNs as previously described (Isci *et al.*, 2011). The pathway names and their graph properties can be found in Supplementary Table S3. For each BN, a dataset of size 50 was generated using BNT with CPTs fitting to the DAGs. The original DAGs were used to obtain distorted prior matrices. In this case, distortion was introduced by adding Gaussian noise to the true DAG’s adjacency matrix *A_T_* to obtain the prior matrix *B*. The distortion rate was calculated using *d* = Fro (*A_T_* – *B*)/Fro(*A_T_*), where Fro(*A*) represents the Frobenius norm of the matrix *A* (da Piedade *et al.*, 2009). The distortion rate was set to be in the [0.0–0.3] range and this range was covered in 0.05 increments rendering seven discrete rates. For each pathway and distortion rate, the synthetic data generation, distortion and structure-learning steps were repeated five times both using the proposed method based on information priors and the likelihood based standard methods. In Figure 4, we represent the average AUC values as a function of the introduced distortion rates. For all iterations, learnt DAGs with informative priors had higher AUCs (between 0.9 and 1) compared to the AUCs (between 0.5 and 0.6) for DAGs learnt with flat priors. The proposed method showed less variation in its performance measure compared to the standard methods. As the distortion level was increased, the difference between the mean AUC values of DAGs learnt with informative prior and flat prior had a tendency to decrease.

### 3.5 Simulated pathway data

We used the same 23 KEGG pathways used in the previous step to generate simulated gene expression data. SynTReN v1.12 was used to generate the signal levels for the genes in each of the 23 pathways with 10 control and 10 test samples and ∼10% background noise (Van den Bulcke *et al.*, 2006). The input data for structure learning was obtained as previously described (Isci *et al.*, 2011). Briefly, columns represent genes in the pathway and rows represent observations. Each row (observation) is obtained by the fold change values of the genes between one pair of control and test samples. The input matrix consisted of 100 observations (10 control × 10 test) and reflected the distribution of fold change values between the two classes of samples. This matrix was discretized into three levels using *k*-means clustering (Li *et al.*, 2010). The inferred DAGs using prior knowledge (proposed method) and unifrom prior knowledge (flat prior, standard methods) were compared to the original pathway structures using AUC values. This process was repeated five times for each pathway.

**Fig. 5.**
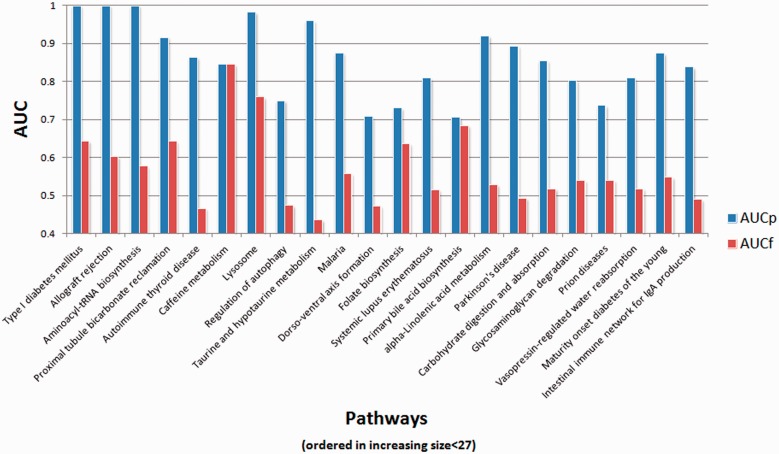
Average AUC values for 23 KEGG pathways based on simulated gene expression data. Proposed method using informative priors (AUCp) and standard methods using flat priors (AUCf) were compared. For each pathway, simulated gene-expression values were used after some data preprocessing to reverse engineer the original DAG. In the simulations, 10 test and 10 control samples, yielding a dataset size of 100, were used. Five simulated datasets per pathway were produced

When the proposed method was employed, the BNP was instantiated for each gene pair in the given pathway to obtain the GI probability for the pair omitting the evidence from the KEGG information priors. These values made up the prior information matrix, *B*. During the instantiation, the evidence vector used composed of existing evidence information for the gene pair in the databases and the microarray correlation value calculated by the input GI data. This exemplifies the utility of the proposed method in which one can build interaction networks based on different evidence types originating from the performed experimental data. The BNP workflow then collates this observed information with the distilled structure obtained from external knowledge bases to infer the GI probability for a pair of genes. The results for the AUC values between predicted and true DAGs for the 23 KEGG pathways using simulated GI data are shown in Figure 5. The proposed method dramatically surpassed classical structure learning methods where the AUC values for the DAGs found using the proposed method, on average, were 30% higher. The average AUC value for the proposed method was 86%. The improvement introduced by BNP shows the value of incorporating existing external knowledge when reverse engineering GI networks from noisy GI data.

### 3.6 Real microarray data

**Fig. 6.**
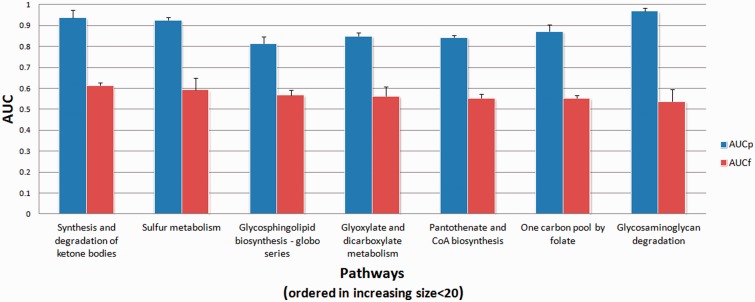
AUC values for the seven KEGG pathways known to be active in Renal Cell Cancer (RCC). For each of the seven pathways real microarray data was used to obtain the observation matrices used in the structure learning process. Proposed method using informative priors (AUCp) and likelihood based methods using flat priors (AUCf) were used to compare the learned networks with the original KEGG pathways

We tested the proposed method using real GI data obtained from Renal Cell Cancer (RCC) and Normal samples as deposited in NCBI’s GEO database with accession numbers GSE 11024 (Kort et al., 2008) and GSE 8271 (Koeman et al., 2008). Input data was obtained as previously described (Isci et al., 2011). Briefly, MAS 5.0 normalized data was used and IDs in the array platform that correspond to a given node in a given pathway were pooled and summarized as one representative signal value using one-step Tukey’s bi-weight algorithm (Hoaglin et al., 2000). Generation of the observation matrix to be used in the structure learning process for a given pathway and incorporation of BNP were carried out as explained in the previous subsection. We attempted at finding seven KEGG pathways shown to be important in RCC (Isci et al., 2011) using the expression values of the genes in these pathways from the two real RCC microarray datasets. The AUC values for the predicted and true pathways using the proposed method and likelihood scoring based methods are shown in Figure 6. In all seven cases, the proposed method found the underlying KEGG pathway with greater accuracy. The average AUC values for the proposed and existing methods were 89% and 57%, respectively. In Supplementary Figure S5, we show the GI network found using the proposed method for the genes in the ‘glycosaminoglycan degradation’ pathway. The comparison of this network with the true KEGG pathway (hsa00531) shows that >95% of the edges that exist in the true pathway are correctly found in the reconstructed network. The proposed method inserted six edges that did not exist in the true pathway. However, as biological pathways may be incomplete, these inserted edges have the potential to suggest interactions that are yet to be discovered and should not be regarded as real false positives.

## 4 DISCUSSION

In this article, we describe a framework to incorporate multiple sources of prior knowledge, regardless of its type, into Bayesian network learning. In several studies, the use of prior biological knowledge of the GI network in conjunction with GI data has been suggested to improve the fidelity of network reconstruction. However, existing methods fail to rigorously harness and use the existing wide range of biological information. The proposed BNP model makes inferences about interactions between gene pairs. The model is instantiated each time with the given experimental data to infer whether the gene pair is related or not, represented by a prediction value between 0 and 1. A prior knowledge matrix is populated with prediction values for all combinations of gene pairs. Using a proposed energy and informative prior function, the prior knowledge is utilized in learning network structure with the Greedy Search algorithm in the BN framework. The goal on these applications is to construct gene networks from GI data and a list of genes of interest. We tested the sensitivity our prior model to its parameters and analyzed the performance of the posterior probability scoring with informative priors against scoring with flat priors. Our BNP model incorporating selective evidence types rendered an accuracy of >90% when estimating if two genes interact given external biological knowledge. This informative prior formula is integrated into the greedy search algorithm to learn Bayesian networks. It was shown that the proposed method was able to infer real pathways with high AUC values, using both synthetic and real GI data.

The proposed framework can be extended to analyze time series gene expression data using Dynamic BNs. This can be achieved through a straightforward application where the calculated *P*(*G*) using BNP is incorporated in dynamic network-learning methods.

Bayesian structure learning algorithms and the improved algorithms described in this article have certain limitations in terms of the size of the network to apply to. Any biological pathway may not work alone but function as part of a large atlas. Therefore, inferring large GI networks (atlas) from data is an important but challenging task. The proposed method is applicable to this problem when the network to be learned can be decomposed into a modular structure.

*Funding*: Scientific and Technological Research Council of Turkey (TUBITAK) [grant 111E042 to H.H.O.].

*Conflict of Interest*: None declared.

## Supplementary Material

Supplementary Data
