# Supplementary material for: Bayesian network prior: network analysis of biological data using external knowledge
Source: Bioinformatics. 2013 Nov 9;30(6):860–7. doi: 10.1093/bioinformatics/btt643 (PMC3957076; doi:10.1093/bioinformatics/btt643)
Supplement: Supplementary Data [file supp_30_6_860__index.html]

Bayesian Network Prior: Network Analysis of Biological Data Using External Knowledge — Bayesian network prior: network analysis of biological data using external knowledge — Bayesian network prior: network analysis of biological data using external knowledge — Supplementary Data 

# Bayesian network prior: network analysis of biological data using external knowledge

## Supplementary Data

files

**Files in this Data Supplement:**

- Supplementary Data - doc file
