# Supplementary material for: Bayesian network prior: network analysis of biological data using external knowledge
Source: Bioinformatics. 2013 Nov 9;30(6):860–7. doi: 10.1093/bioinformatics/btt643 (PMC3957076; doi:10.1093/bioinformatics/btt643)
Supplement: Supplementary Data [file supp_btt643_OTU_Supplementary_Information.doc]

**Supplementary Information**

**for**

**Bayesian Network Prior: Network Analysis of Biological Data Using External Knowledge**

**by**

**Senol Isci, Haluk Dogan, Cengizhan Ozturk, Hasan H. Otu**

This Supplementary Information document includes Supplementary Figures and Tables that are relevant to the results, discussions, and conclusions of the main manuscript.

**Supplementary Figures**

**
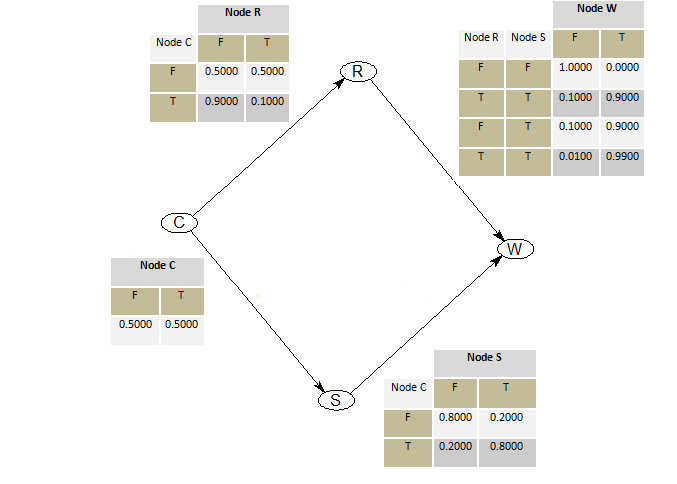
**

**Figure S1.** Topology and the conditional probability tables used for the Sprinkler BN.


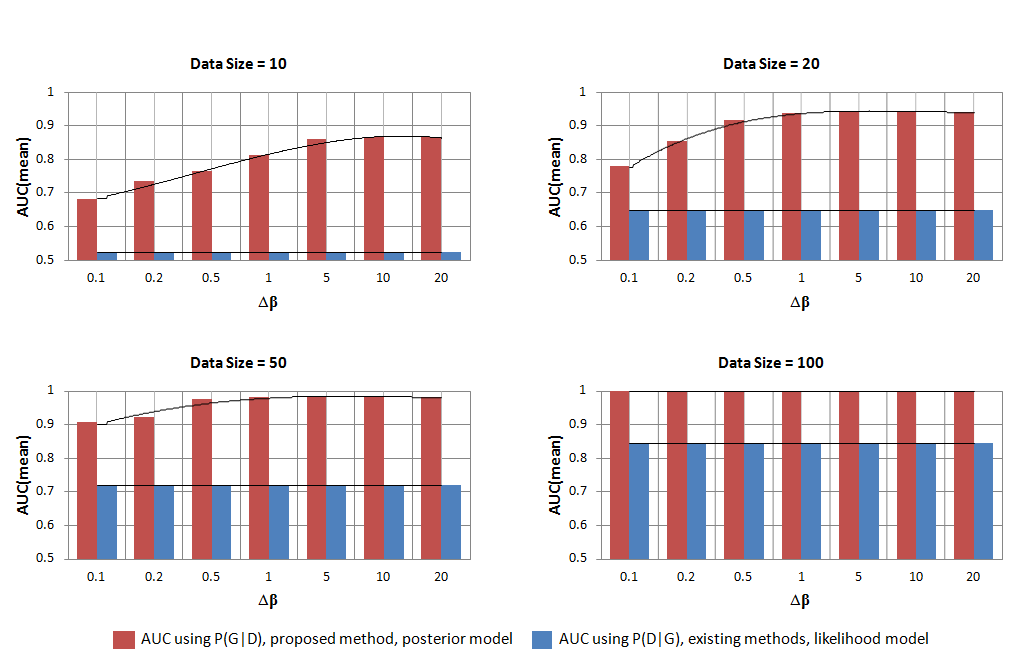


**Figure S2.** Performance of the proposed method as a function of the hyperparameter β. Data set sizes of 10, 20, 50, and 100 were generated for the Sprinkler BN and Area Under the Curve (AUC) values for the proposed (shown in red) and standard (shown in blue) methods were compared as a function of ∆β = β_H_ – β_L_. Each bar in the graphs represents the average AUC value of 50 simulations.


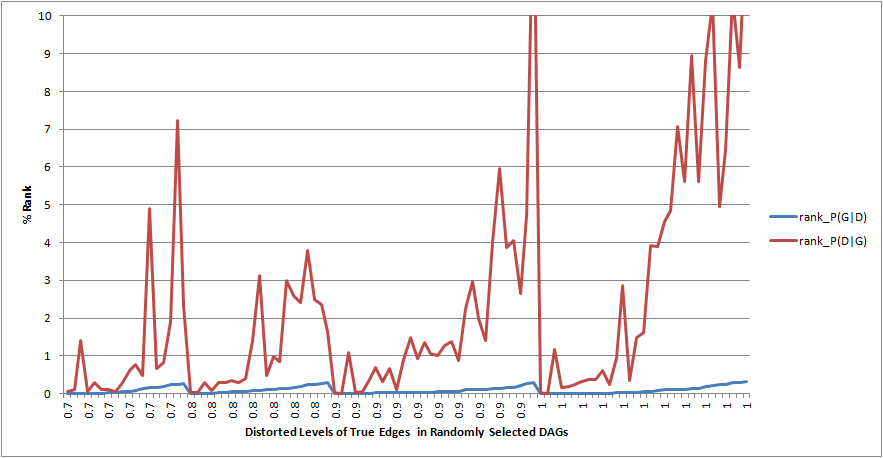


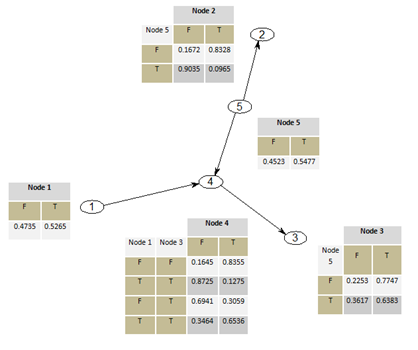
**Figure S3.** Percent rank of randomly chosen 5-node BNs using the proposed, P(G|D), and standard, P(D|G), scoring schemes. The prior matrix B used in the proposed method is distorted so that the true edges have a probability of 0.7, 0.8, 0.9, or 1.0. Percent rank depicts the scaled rank of the true DAG among all possible 5-node DAGs based on the two scoring methods.

**Figure S4.** Topology and the conditional probability tables used for the 5-node BN.

**
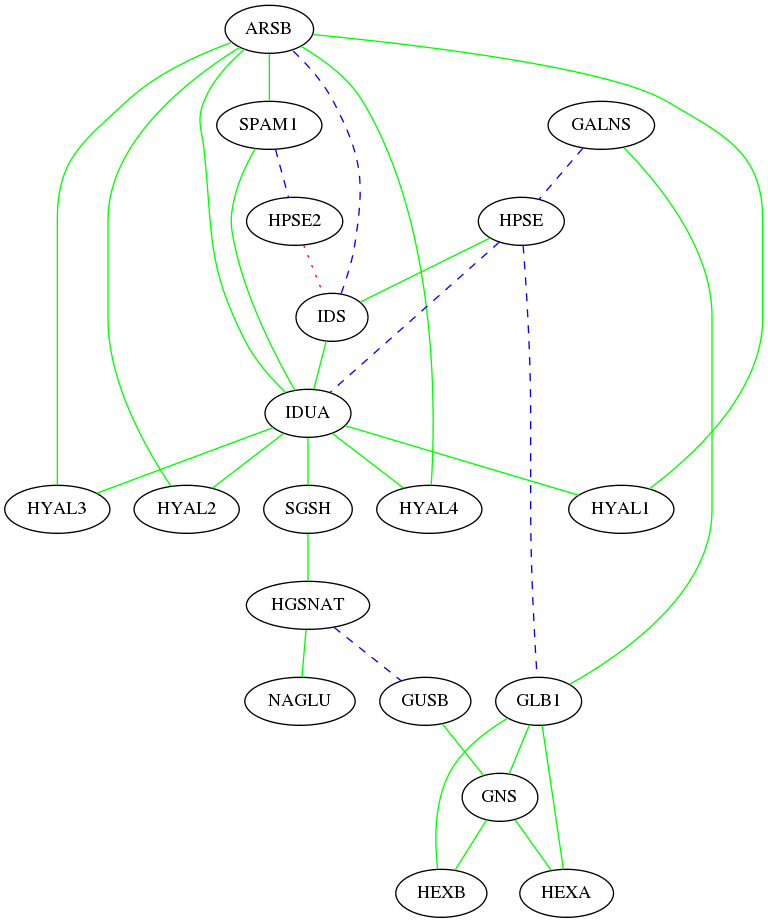
**

**Figure S5.** Reconstructed gene interaction network for the KEGG pathway "glycosaminoglycan degradation" (hsa00531) using the proposed method based on real RCC microarray data. The green links are matching links between the KEGG pathway and the learnt DAG. Red dotted links are missing in the learnt DAG but exist in the KEGG pathway. Blue dotted links are inserted edges that exist in the learnt DAG but not in the KEGG pathway.

**Supplementary Tables**

**Table S1**. Evidence types used in building the Bayesian Network Prior (BNP). For a gene pair, any combination of these evidence types can be presented to the proposed system to infer the gene interaction network. The descriptions are obtained from appropriate databases.

| **Evidence Type** | **Description** |
| --- | --- |
| Affinity Capture-MS | An interaction is inferred when a "bait" protein is affinity captured from cell extracts by either polyclonal antibody or epitope tag and the associated interaction partner is identified by mass spectrometric methods. |
| Biochemical Activity | An interaction is inferred from the biochemical effect of one protein upon another, for example, GTP-GDP exchange activity or phosphorylation of a substrate by a kinase. The "bait" protein executes the activity on the substrate "hit" protein. |
| Reconstituted Complex | An interaction is detected between purified proteins in vitro. |
| Pathway | An interaction is observed in at least two of the following three pathway databases: KEGG, NCI/NATURE, and Reactome. |
| Far Western | An interaction is detected between a protein immobilized on a membrane and a purified protein probe. |
| Co-purification | An interaction is inferred from the identification of two or more protein subunits in a purified protein complex, as obtained by classical biochemical fractionation or affinity purification and one or more additional fractionation steps. |
| Two-hybrid / TF Binding Site Localization | Bait protein expressed as a DNA binding domain (DBD) fusion and prey expressed as a transcriptional activation domain (TAD) fusion and interaction measured by reporter gene activation. |
| Phenotypic Suppression | A genetic interaction is inferred when mutation or over expression of one gene results in suppression of any phenotype (other than lethality/growth defect) associated with mutation or over expression of another gene. |
| FRET | An interaction is inferred when close proximity of interaction partners is detected by fluorescence resonance energy transfer between pairs of fluorophore-labeled molecules, such as occurs between CFP (donor) and YFP (acceptor) fusion proteins. |
| Affinity Capture-Western | An interaction is inferred when a bait protein affinity captured from cell extracts by either polyclonal antibody or epitope tag and the associated interaction partner identified by Western blot with a specific polyclonal antibody or second epitope tag. This category is also used if an interacting protein is visualized directly by dye stain or radioactivity. Note that this differs from any co-purification experiment involving affinity capture in that the co-purification experiment involves at least one extra purification step to get rid of potential contaminating proteins. |
| Co-localization | An interaction is inferred from co-localization of two proteins in the cell, including co-dependent association of proteins with promoter DNA in chromatin immunoprecipitation experiments. |
| Protein-peptide | An interaction is detected between a protein and a peptide derived from an interaction partner. This includes phage display experiments. |
| Co-crystal Structure | Interaction directly demonstrated at the atomic level by X-ray crystallography. Also used for NMR or Electron Microscopy (EM) structures. If a structure is demonstrated between 3 or more proteins, one is chosen as the bait and binary interactions are recorded between that protein and the others. |
| Affinity Capture-Luminescence | An interaction is inferred when a bait protein, tagged with luciferase, is enzymatically detected in immunoprecipitates of the prey protein as light emission. The prey protein is affinity captured from cell extracts by either polyclonal antibody or epitope tag. |
| Synthetic Growth Defect | A genetic interaction is inferred when mutations in separate genes, each of which alone causes a minimal phenotype, result in a significant growth defect under a given condition when combined in the same cell. |
| Phenotypic Enhancement | A genetic interaction is inferred when mutation or overexpression of one gene results in enhancement of any phenotype (other than lethality/growth defect) associated with mutation or over expression of another gene. |
| Co-fractionation | Interaction inferred from the presence of two or more protein subunits in a partially purified protein preparation. If co-fractionation is demonstrated between 3 or more proteins, one is chosen as the bait and binary interactions are recorded between that protein and the others. |
| Synthetic Rescue | A genetic interaction is inferred when mutations or deletions of one gene rescues the lethality or growth defect of a strain mutated or deleted for another gene. |
| Microarray Correlation | An interaction is inferred if the centered Pearson’s correlation is over 0.98. |

**Table S2**. Ranking of top 10 scoring DAGs for the Sprinkler Network using prior matrix, B, different than the true adjacency matrix. Data set size = 1000. The correct DAG number is 504. DAGs 491, 503, and 504 belong to the same Markov Equivalence Class.

| TRUE ADJ. MAT | **C** | **R** | **S** | **W** |
| --- | --- | --- | --- | --- |
| **C** | 0 | 1 | 1 | 0 |
| **R** | 0 | 0 | 0 | 1 |
| **S** | 0 | 0 | 0 | 1 |
| **W** | 0 | 0 | 0 | 0 |
|  |  |  |  |  |
|  |  | **TRIAL # 1** |  |  |
| PRIOR ADJ. MAT | **C** | **R** | **S** | **W** |
| **C** | 0 | 0.98 | 0.98 | 0 |
| **R** | 0 | 0 | 0 | 0.98 |
| **S** | 0 | 0 | 0 | 0.98 |
| **W** | 0 | 0 | 0 | 0 |
|  |  |  |  |  |
|  | **DAG #** | **log [P(D|G)]** | **log [P(G|D)]** |  |
|  | 504 | -1926.649 | -1931.473 |  |
|  | 491 | -1926.649 | -1960.762 |  |
|  | 503 | -1926.649 | -1960.762 |  |
|  | 533 | -1932.315 | -1967.766 |  |
|  | 506 | -1932.663 | -1968.119 |  |
|  | 518 | -1932.663 | -1968.119 |  |
|  | 524 | -1932.315 | -1997.760 |  |
|  | 532 | -1932.315 | -1997.760 |  |
|  | 501 | -1932.663 | -1998.119 |  |
|  | 517 | -1932.663 | -1998.119 |  |
|  |  |  |  |  |
|  |  | **TRIAL # 2** |  |  |
| PRIOR ADJ. MAT | **C** | **R** | **S** | **W** |
| **C** | 0 | 0.6 | 0.6 | 0 |
| **R** | 0 | 0 | 0 | 0.6 |
| **S** | 0 | 0 | 0 | 0.6 |
| **W** | 0 | 0 | 0 | 0 |
|  | **DAG #** | **log [P(D|G)]** | **log [P(G|D)]** |  |
|  | 504 | -1926.649 | -2026.274 |  |
|  | 491 | -1926.649 | -2065.391 |  |
|  | 503 | -1926.649 | -2065.391 |  |
|  | 533 | -1932.315 | -2098.177 |  |
|  | 506 | -1932.663 | -2098.554 |  |
|  | 518 | -1932.663 | -2098.554 |  |
|  | 502 | -2041.320 | -2119.820 |  |
|  | 524 | -1932.315 | -2139.100 |  |
|  | 532 | -1932.315 | -2139.100 |  |
|  | 501 | -1932.663 | -2139.484 |  |
|  |  |  |  |  |
|  |  | **TRIAL # 3** |  |  |
| PRIOR ADJ. MAT | **C** | **R** | **S** | **W** |
| **C** | 0 | 0.3 | 0.3 | 0 |
| **R** | 0 | 0 | 0 | 0.3 |
| **S** | 0 | 0 | 0 | 0.3 |
| **W** | 0 | 0 | 0 | 0 |
|  |  |  |  |  |
|  | **DAG #** | **log [P(D|G)]** | **log [P(G|D)]** |  |
|  | 504 | -1926.649 | -2105.510 |  |
|  | 491 | -1926.649 | -2125.954 |  |
|  | 503 | -1926.649 | -2125.954 |  |
|  | 533 | -1932.315 | -2181.075 |  |
|  | 502 | -2041.320 | -2181.340 |  |
|  | 506 | -1932.663 | -2181.467 |  |
|  | 518 | -1932.663 | -2181.467 |  |
|  | 485 | -1969.939 | -2194.894 |  |
|  | 484 | -2041.320 | -2202.370 |  |
|  | 524 | -1932.315 | -2202.468 |  |
|  |  |  |  |  |
|  |  | **TRIAL # 4** |  |  |
| PRIOR ADJ. MAT | **C** | **R** | **S** | **W** |
| **C** | 0 | 0 | 0.98 | 0 |
| **R** | 0 | 0 | 0 | 0.98 |
| **S** | 0 | 0 | 0 | 0.98 |
| **W** | 0 | 0 | 0 | 0 |
|  |  |  |  |  |
|  | **DAG #** | **log [P(D|G)]** | **log [P(G|D)]** |  |
|  | 504 | -1926.649 | -2026.274 |  |
|  | 491 | -1926.649 | -2065.391 |  |
|  | 503 | -1926.649 | -2065.391 |  |
|  | 533 | -1932.315 | -2098.177 |  |
|  | 506 | -1932.663 | -2098.554 |  |
|  | 518 | -1932.663 | -2098.554 |  |
|  | 502 | -2041.320 | -2119.820 |  |
|  | 524 | -1932.315 | -2139.100 |  |
|  | 532 | -1932.315 | -2139.100 |  |
|  | 501 | -1932.663 | -2139.484 |  |
|  |  |  |  |  |
|  |  | **TRIAL # 5** |  |  |
| PRIOR ADJ. MAT | **C** | **R** | **S** | **W** |
| **C** | 0 | 0 | 0.98 | 0 |
| **R** | 0 | 0 | 0 | 0.6 |
| **S** | 0 | 0 | 0 | 0.6 |
| **W** | 0 | 0 | 0 | 0 |
|  |  |  |  |  |
|  | **DAG #** | **log [P(D|G)]** | **log [P(G|D)]** |  |
|  | 504 | -1926.649 | -2040.502 |  |
|  | 503 | -1926.649 | -2040.502 |  |
|  | 502 | -2041.320 | -2094.534 |  |
|  | 491 | -1926.649 | -2105.510 |  |
|  | 532 | -1932.315 | -2113.062 |  |
|  | 533 | -1932.315 | -2113.062 |  |
|  | 506 | -1932.663 | -2113.441 |  |
|  | 517 | -1932.663 | -2113.441 |  |
|  | 518 | -1932.663 | -2113.441 |  |
|  | 505 | -2004.043 | -2122.471 |  |
|  |  |  |  |  |
|  |  | **TRIAL # 6** |  |  |
| PRIOR ADJ. MAT | **C** | **R** | **S** | **W** |
| **C** | 0 | 0 | 0.1 | 0 |
| **R** | 0 | 0 | 0 | 0.1 |
| **S** | 0 | 0 | 0 | 0.1 |
| **W** | 0 | 0 | 0 | 0 |
|  |  |  |  |  |
|  | **DAG #** | **log [P(D|G)]** | **log [P(G|D)]** |  |
|  | 504 | -1926.649 | -2167.630 |  |
|  | 503 | -1926.649 | -2167.630 |  |
|  | 491 | -1926.649 | -2174.679 |  |
|  | 485 | -1969.939 | -2223.543 |  |
|  | 502 | -2041.320 | -2223.667 |  |
|  | 484 | -2041.320 | -2230.827 |  |
|  | 165 | -1982.210 | -2244.677 |  |
|  | 532 | -1932.315 | -2246.081 |  |
|  | 533 | -1932.315 | -2246.081 |  |
|  | 506 | -1932.663 | -2246.484 |  |
|  |  |  |  |  |
|  |  | **TRIAL # 7** |  |  |
| PRIOR ADJ. MAT | **C** | **R** | **S** | **W** |
| **C** | 0.5 | 0.98 | 0.98 | 0.5 |
| **R** | 0.5 | 0.5 | 0.5 | 0.98 |
| **S** | 0.5 | 0.5 | 0.5 | 0.98 |
| **W** | 0.5 | 0.5 | 0.5 | 0.5 |
|  |  |  |  |  |
|  | **DAG #** | **log [P(D|G)]** | **log [P(G|D)]** |  |
|  | 504 | -1926.649 | -1931.473 |  |
|  | 491 | -1926.649 | -1960.762 |  |
|  | 503 | -1926.649 | -1960.762 |  |
|  | 533 | -1932.315 | -1967.766 |  |
|  | 506 | -1932.663 | -1968.119 |  |
|  | 518 | -1932.663 | -1968.119 |  |
|  | 524 | -1932.315 | -1997.760 |  |
|  | 532 | -1932.315 | -1997.760 |  |
|  | 501 | -1932.663 | -1998.119 |  |
|  | 517 | -1932.663 | -1998.119 |  |
|  |  |  |  |  |
|  |  | **TRIAL # 8** |  |  |
| PRIOR ADJ. MAT | **C** | **R** | **S** | **W** |
| **C** | 0.5 | 0.6 | 0.6 | 0.5 |
| **R** | 0.5 | 0.5 | 0.5 | 0.6 |
| **S** | 0.5 | 0.5 | 0.5 | 0.6 |
| **W** | 0.5 | 0.5 | 0.5 | 0.5 |
|  |  |  |  |  |
|  | **DAG #** | **log [P(D|G)]** | **log [P(G|D)]** |  |
|  | 504 | -1926.649 | -2026.274 |  |
|  | 491 | -1926.649 | -2032.725 |  |
|  | 503 | -1926.649 | -2032.725 |  |
|  | 533 | -1932.315 | -2064.858 |  |
|  | 506 | -1932.663 | -2065.229 |  |
|  | 518 | -1932.663 | -2065.229 |  |
|  | 524 | -1932.315 | -2071.466 |  |
|  | 532 | -1932.315 | -2071.466 |  |
|  | 501 | -1932.663 | -2071.838 |  |
|  | 517 | -1932.663 | -2071.838 |  |
|  |  |  |  |  |
|  |  | **TRIAL # 9** |  |  |
| PRIOR ADJ. MAT | **C** | **R** | **S** | **W** |
| **C** | 0.5 | 0.5 | 0.98 | 0.5 |
| **R** | 0.5 | 0.5 | 0.5 | 0.98 |
| **S** | 0.5 | 0.98 | 0.5 | 0.98 |
| **W** | 0.5 | 0.5 | 0.5 | 0.5 |
|  |  |  |  |  |
|  | **DAG #** | **log [P(D|G)]** | **log [P(G|D)]** |  |
|  | 503 | -1926.649 | -1960.762 |  |
|  | 504 | -1926.649 | -1960.762 |  |
|  | 506 | -1932.663 | -1968.119 |  |
|  | 491 | -1926.649 | -1990.644 |  |
|  | 532 | -1932.315 | -1997.760 |  |
|  | 533 | -1932.315 | -1997.760 |  |
|  | 495 | -1932.663 | -1998.119 |  |
|  | 501 | -1932.663 | -1998.119 |  |
|  | 517 | -1932.663 | -1998.119 |  |
|  | 518 | -1932.663 | -1998.119 |  |

**Table S3.** Several graph parameters of KEGG pathways used to generate simulated data sets.

| **Pathway** | **size**  **(nodes)** | **order**  **(edges)** | **density** | **max degree** | **avg. degree** |
| --- | --- | --- | --- | --- | --- |
| D-Glutamine and D-glutamate metabolism | 4 | 5 | 0.833 | 3 | 2.5 |
| Type I diabetes mellitus | 5 | 3 | 0.3 | 2 | 1.2 |
| Allograft rejection | 7 | 4 | 0.190 | 2 | 1.142 |
| Aminoacyl-tRNA biosynthesis | 7 | 5 | 0.238 | 3 | 1.428 |
| Autoimmune thyroid disease | 7 | 4 | 0.190 | 2 | 1.142 |
| Caffeine metabolism | 7 | 6 | 0.285 | 6 | 1.714 |
| Proximal tubule bicarbonate reclamation | 7 | 6 | 0.285 | 2 | 1.714 |
| Lysosome | 8 | 7 | 0.25 | 7 | 1.75 |
| Mineral absorption | 8 | 6 | 0.214 | 5 | 1.5 |
| Regulation of autophagy | 8 | 6 | 0.214 | 5 | 1.5 |
| Sulfur relay system | 8 | 8 | 0.286 | 5 | 2 |
| Fat digestion and absorption | 9 | 16 | 0.444 | 5 | 3.555 |
| Taurine and hypotaurine metabolism | 9 | 18 | 0.5 | 6 | 4 |
| Malaria | 11 | 7 | 0.127 | 3 | 1.273 |
| Arrhythmogenic right ventricular cardiomyopathy (ARVC) | 12 | 10 | 0.152 | 4 | 1.666 |
| Dorso-ventral axis formation | 12 | 14 | 0.212 | 5 | 2.333 |
| Folate biosynthesis | 13 | 11 | 0.141 | 5 | 1.692 |
| Glycosphingolipid biosynthesis - globo series | 13 | 66 | 0.846 | 11 | 10.154 |
| Sulfur metabolism | 13 | 24 | 0.308 | 12 | 3.692 |
| Systemic lupus erythematosus | 14 | 30 | 0.330 | 8 | 4.286 |
| Glyoxylate and dicarboxylate metabolism | 15 | 21 | 0.2 | 5 | 2.8 |
| Primary bile acid biosynthesis | 16 | 53 | 0.442 | 13 | 6.625 |
| Pantothenate and CoA biosynthesis | 17 | 39 | 0.287 | 8 | 4.588 |
